# Onset of asthma‐like symptoms in children with lower respiratory tract infections

**DOI:** 10.1002/jcla.23227

**Published:** 2020-02-10

**Authors:** Song Mao, Li Fang, Liangxia Wu, Wenjing Shi, Min Xu

**Affiliations:** ^1^ Department of Pediatrics Shanghai Jiao Tong University Affiliated Sixth People's Hospital Shanghai China; ^2^ China Hospital Development Institute Shanghai Jiao Tong University Shanghai China

**Keywords:** asthma‐like symptoms, children, lower respiratory tract infections, risk

## Abstract

**Background:**

Asthma‐like symptoms (ALS) often occur among children with lower respiratory tract infections (LRTIs). We aimed to determine the potential risk factors for ALS onset in LRTIs children.

**Methods:**

A total of 102 LRTIs with ALS and 474 without ALS were enrolled. The relative risk (RR) was used to test the influence of the clinical factors on the ALS risk. We compared the differences of birth data, wheezing history, disease severity, inflammatory markers, infectious pathogens, allergic markers, cardiac, liver, and kidney injury markers between LRTIs with and without ALS onset. Receiver operating curve (ROC) analysis was applied to determine the predictive value of various markers in the ALS risk in LRTIs. Multivariate logistic regression analysis was performed to evaluate the association between various clinical and laboratory parameters and ALS onset in LRTIs.

**Results:**

The RRs of boys/girls ratio and wheezing history for ALS compared with non‐ALS was 1.263 and 2.850, respectively (*P* = .026, <10^−4^). There were significant differences of age, WBC, PLT, EOS, and CK between LRTIs with and without ALS onset (*P* = .004, .041, .006, .049, and .035). ROC analysis showed that significant associations between the parameters of age, WBC, and PLT and ALS risk among LRTIs were observed. Multivariate logistic regression analysis showed that the clinical and laboratory parameters were not independently associated with the risk of ALS onset among LRTIs.

**Conclusions:**

Lower age, male, inflammation, and allergic state were risk factors for ALS onset in LRTIs. Comprehensive monitoring and evaluation of these factors may be helpful for ALS prevention.

**Quick look**

**Current knowledge**

1. ALS was a common symptom in children with LRTIs.
2. A number of children with ALS onset were prone to develop asthma.

**What this paper contributes to our knowledge**

1. Lower age, male, inflammation, and allergic state were risk factors for ALS onset in LRTIs.
2. The potential risk factors were not independently associated with ALS susceptibility in LRTIs.
3. Comprehensive monitoring and evaluation of the risk factors were helpful for the ALS prevention.

## 1 INTRODUCTION

Asthma is the most common chronic disease of children.^1^ Increased incidence of asthma leads to significant morbidity and mortality. Regional and individual variations in the asthma prevalence indicate the existence of different risk factors.

Genetic factors, environmental exposures, pathogens infections, and interactions between these factors may affect the susceptibility to asthma.^2^, ^3^, ^4^, ^5^ On the other hand, a number of children lack the typical clinical presentations, particularly in the cases aged less than 6 years.^6^ These children are difficult to be evaluated, often presenting with wheezing and breathlessness, regarded as asthma‐like symptoms (ALS). Although many ALS cases were self‐limited, a number of children with ALS were prone to develop asthma. In this sense, early identification of risk factors for ALS onset in children seems of great clinical implications.

During the past years, studies focusing on the ALS in children showed that the rates and severity of ALS increased.^7^ Obesity is an important risk factor for asthma and wheeze.^8^ Elevated BMI was associated with a greater prevalence of wheezing and eczema.^9^ Research efforts have been focused on the tools to identify who wheeze will progress to develop asthma. Bronchial airway chronic inflammation is the hallmark of asthma, which is characterized by the imbalance of oxidative stress and antioxidant defenses.^9^ Airway hyperresponsiveness is likely to induce the recurrence of ALS and asthma.^10^ Notably, asthma is often preceded by recurrent episodes of troublesome lung symptoms. Many lower respiratory tract infections (LRTIs) patients are likely to be complicated with ALS, while some are not. Previous studies showed that genetic factors, environmental pollution, BMI, and gender were associated with asthma‐like disease.^5^, ^11^, ^12^, ^13^ However, the influence of the clinical and laboratory parameters, such as birth data, on the risk of ALS remains elusive.

To have an in‐depth understanding of this issue, we determined to perform a prospective study of the differences of various indexes, including birth data, wheezing history, disease severity, inflammatory markers, infectious pathogens, allergic markers, cardiac, liver, and kidney injury markers between LRTIs with and without ALS onset. Predictive value of various markers in the ALS risk in LRTIs was tested. Multivariate logistic regression analysis was conducted to assess the association between the clinical and laboratory parameters and the risk of ALS among LRTIs cases.

## 2 MATERIALS AND METHODS

### 2.1 Patient population

We conducted a prospective study of the potential risk factors for ALS onset among children with LRTIs. We recruited the LRTIs subjects between January 2016 and August 2018 from the inpatients admitted to the Department of Pediatrics, Shanghai Sixth People's Hospital, China. The enrolled participants' age was between 1 and 14 years. ALS occurred during the course of hospitalization. Patients with systemic diseases that may influence the risk of ALS were excluded. All the guardians of enrolled subjects signed the informed consent. We collected the birth data, wheezing history, age, and gender. The severe LRTIs were defined as the cases were complicated with multi‐system disorders. Onset of ALS was defined as the acute attack of cough, wheezing, and dyspnea. The blood and urine samples were during the first day after admission. The specimens were stored at −70°C. All the guardians of children signed the informed consent which was ratified by the ethic committee.

### 2.2 Pathogens detection

The blood samples were handled according to the manufacturer's instructions. The molecular tests were performed in the clinical laboratory of Shanghai Sixth People's Hospital. Real‐time PCR were applied to test the following pathogens, including mycoplasma pneumoniae (MP), respiratory syncytial virus (RSV), adenovirus, influenza A (FluA), influenza B (FluB), parainfluenza (ParaFlu), Esptein‐Barr (EB), coxsackie (Cox), cytomegalovirus (Cyto), and herpes simplex I+II (Herpes). The results were defined as positive or negative according to the cut‐off value.

### 2.3 Laboratory testing

We determined the indexes of white blood cell (WBC), c‐reactive protein (CRP), and platelet (PLT), eosinophils (EOS) by routine blood test, urine red blood cell (uRBC) by routine urine test, erythrocyte sedimentation rate (ESR), procalcitonin (PCT), blood urea nitrogen (BUN), serum creatinine (Scr), alanine aminotransferase (ALT), aspartate aminotransferase (AST), lactate dehydrogenase (LDH), creatine kinase (CK), and creatine kinase isoenzyme (CKMB) by biochemical automated testing equipment. All the laboratory parameters were tested during the first day after the admission to hospital.

### 2.4 Statistical analysis

The continuous data were expressed as means ± standard deviation (SD). Relative risk (RR) was used test the association between the parameters of boys/girls ratio, preterm (gestation periods <37 weeks), low‐birthweight (<2500 g), cesarean birth, mycoplasma pneumoniae, respiratory virus infections, wheezing history, and severe LRTIs and ALS risk among LRTIs cases. Independent one‐sample t test was applied to determine the differences of age, birthweight, WBC, CRP, PLT, EOS, uRBC, ESR, PCT, BUN, Scr, ALT, AST, LDH, CK, and CKMB between LRTIs with and without ALS onset. ROC analyze were applied to test the predictive value of clinical and laboratory parameters for the risk of ALS onset among children with LRTIs. Multivariate logistic regression analysis was performed to evaluate the relationship between various indexes and ALS onset in LRTIs. All the statistical analyses were conducted by using SPSS version 19. *P* < .05 was considered statistically significant, except where otherwise specified.

## 3 RESULTS

### 3.1 Distribution of various populations among ALS and non‐ALS cases

A total of 102 LRTIs with ALS and 474 without ALS were enrolled in our study. The RR of boys/girls ratio for ALS compared with non‐ALS was 1.263 (*P* = .026). The RR of wheezing history for ALS compared with non‐ALS was 2.850 (*P* < 10^−4^). The RRs of preterm, low‐birthweight, cesarean birth, mycoplasma pneumoniae, respiratory virus infection, and severe LRTIs for ALS compared with non‐ALS was 1.021 (*P* = .942), 0.996 (*P* = .990), 1.145 (*P* = .259), 0.999 (*P* = .995), 1.253 (*P* = .218), and 1.575 (*P* = .108), respectively (Table 1).

**Table 1:** Distribution of various populations among ALS and non‐ALS cases

Groups	ALS (Case/total)	Non‐ALS (Case/total)	Relative risk	*P*
Boys/girls ratio (Total)	72/30	231/243	1.263	.026
Preterm	13/102	59/474	1.021	.942
Low‐birthweight	12/102	56/474	0.996	.990
Cesarean birth	57/102	216/474	1.145	.259
Mycoplasma pneumoniae	49/102	228/474	0.999	.995
Respiratory virus infection	30/102	105/474	1.253	.218
Wheezing history	47/102	59/474	2.850	<10^−4^
Severe LRTI	15/102	42/474	1.575	.108

Abbreviations: ALS, asthma‐like symptoms; LRTI, lower respiratory tract infections; RR, relative risk.

### 3.2 Differences of various parameters between LRTIs with and without ALS

There were significant differences of age, WBC, PLT, EOS, and CK between LRTIs with and without ALS onset (*P* = .004, .041, .006, .049, and .035; Table 2). No marked differences of birthweight, CRP, PCT, ESR, uRBC, BUN, Scr, ALT, AST, LDH, and CKMB were noted between LRTIs with and without ALS onset (Table 2).

**Table 2:** Comparison of clinical and laboratory parameters between ALS and non‐ALS group

Parameter	ALS	Non‐ALS	*P*
Age	3.36 ± 2.44	4.74 ± 2.39	.004
BW	3346.32 ± 946.36	3272.45 ± 617.91	.666
WBC	9.61 ± 5.56	7.36 ± 5.55	.041
CRP	22.62 ± 31.73	19.07 ± 31.99	.563
PLT	295.36 ± 103.38	238.14 ± 102.81	.006
PCT	0.50 ± 1.04	0.55 ± 5.56	.837
ESR	18.67 ± 18.30	21.34 ± 18.32	.452
EOS	0.12 ± 0.13	0.07 ± 0.11	.049
uRBC	5.15 ± 5.94	6.75 ± 7.90	.191
BUN	3.53 ± 0.93	3.34 ± 1.02	.306
Scr	27.06 ± 7.43	29.54 ± 7.89	.093
ALT	16.76 ± 9.94	18.58 ± 37.69	.608
AST	32.36 ± 9.39	35.12 ± 30.28	.355
LDH	322.69 ± 127.99	295.93 ± 89.44	.261
CK	124.51 ± 127.14	96.84 ± 42.78	.035
CKMB	23.87 ± 12.58	23.05 ± 9.37	.728

Abbreviations: ALS, asthma‐like symptoms; ALT, alanine aminotransferase; AST, aspartate aminotransferase; BUN, blood urea nitrogen; BW, birthweight; CK, creatine kinase; CKMB, creatine kinase isoenzyme; CRP, c‐reactive protein; EOS, eosinophils; ESR, erythrocyte sedimentation rate; LDH, lactate dehydrogenase; LRTI, lower respiratory tract infections; PCT, procalcitonin; PLT, platelet; Scr, serum creatinine; uRBC, urine red blood cell; WBC, white blood cell.

### 3.3 ROC analysis of the predictive value of various indexes in the risk of ALS among LRTIs

**Figure 1:**
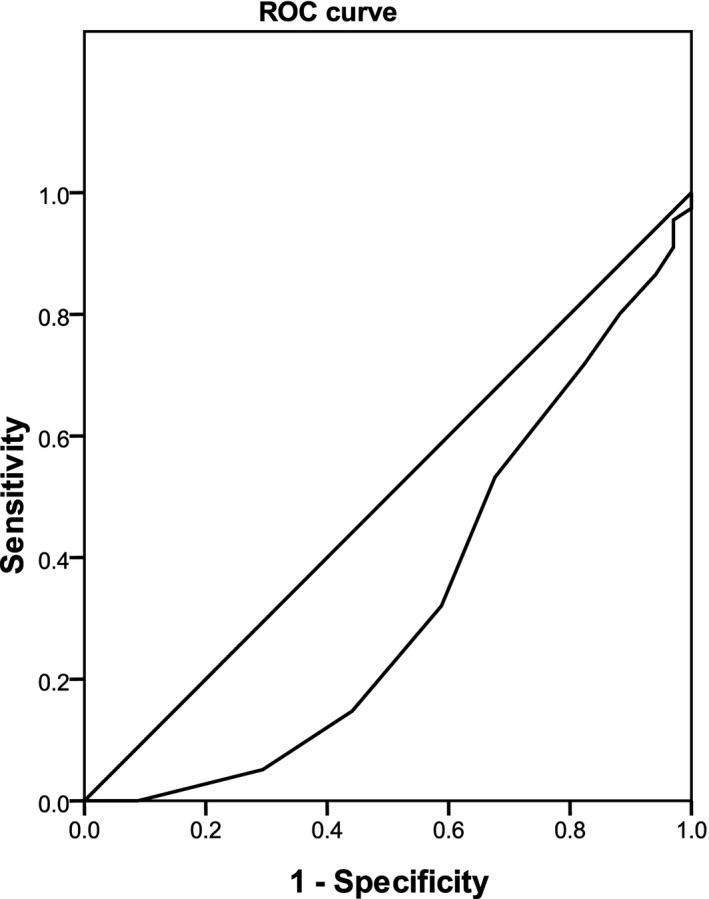
Association between age and asthma‐like symptoms risk in lower respiratory tract infectionss

**Figure 2:**
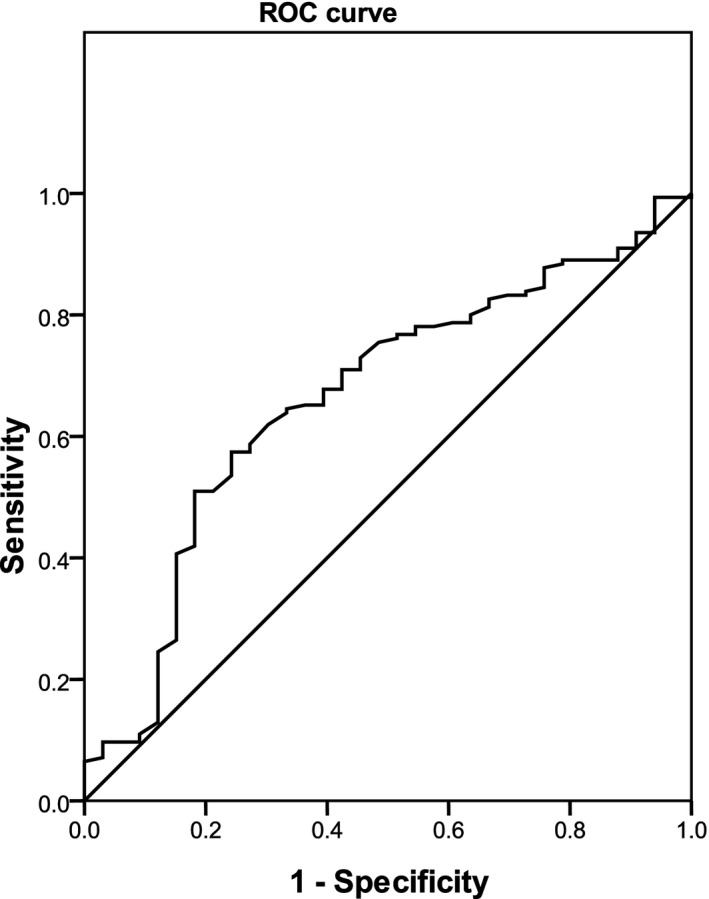
Association between white blood cell and asthma‐like symptoms risk in lower respiratory tract infections

**Figure 3:**
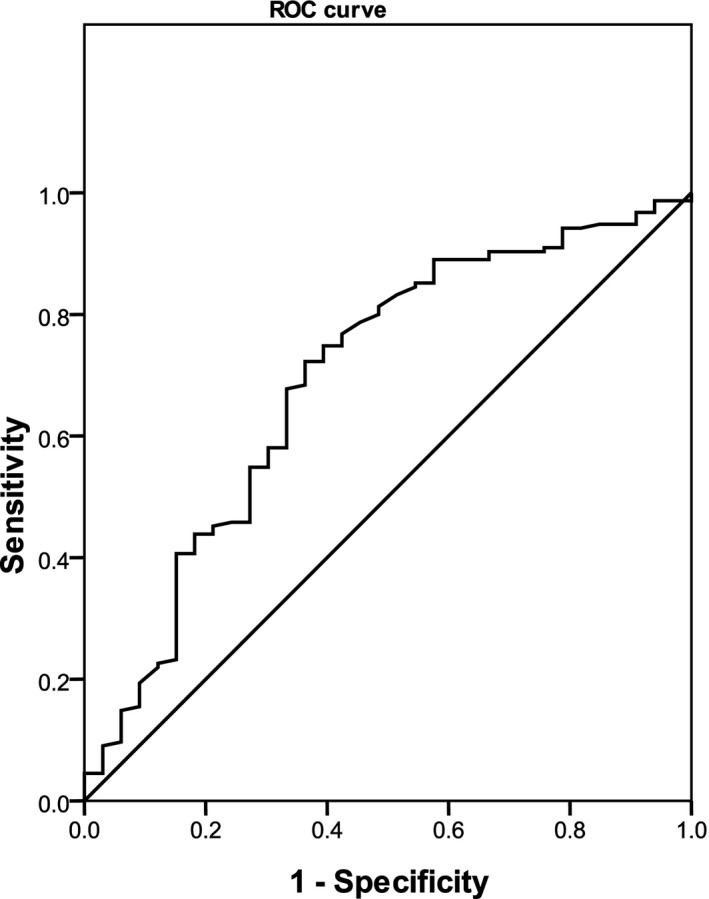
Association between platelet and asthma‐like symptoms risk in lower respiratory tract infections

Significant associations between the parameters of age, WBC, and PLT and ALS risk among LRTIs were observed (Table 3, Figures 1, 2, 3). No marked relationships between birthweight, CRP, PCT, ESR, EOS, uRBC, BUN, Scr, ALT, AST, LDH, CK, and CKMB and ALS onset among LRTIs were noted (Table 3).

**Table 3:** Predictive value of various parameters in the risk of ALS

Parameter	ROC area	95% CI	*P*
Age	0.651	0.534‐0.768	.009
BW	0.549	0.445‐0.654	.393
WBC	0.335	0.228‐0.442	.004
CRP	0.415	0.306‐0.525	.142
PLT	0.316	0.202‐0.430	.001
PCT	0.540	0.418‐0.661	.494
ESR	0.554	0.446‐0.661	.352
EOS	0.448	0.315‐0.581	.369
uRBC	0.555	0.439‐0.670	.341
BUN	0.405	0.292‐0.519	.101
Scr	0.585	0.475‐0.695	.141
ALT	0.408	0.310‐0.507	.111
AST	0.486	0.373‐0.600	.814
LDH	0.430	0.318‐0.542	.225
CK	0.510	0.403‐0.618	.858
CKMB	0.501	0.386‐0.617	.983

Abbreviation: ROC, receiver operating characteristic curve.

### 3.4 Multivariate logistic regression analysis of the relationship between various indexes and ALS onset in LRTIs

Multivariate logistic regression analysis showed that no marked associations were observed between age, birthweight, WBC, PLT, CRP, PCT, ESR, EOS, uRBC, BUN, Scr, ALT, AST, LDH, CK, and CKMB and ALS risk among LRTIs (Table 4), which indicated these clinical and laboratory parameters were not independently associated with the risk of ALS onset among LRTIs.

**Table 4:** Multivariate logistic regression analysis of the parameters and ALS risk

Index	*P*	OR	95%CI
Age	.135	1.294	0.923‐1.815
BW	.631	1.000	0.999‐1.001
WBC	.555	0.973	0.887‐1.066
CRP	.234	0.987	0.967‐1.008
PLT	.215	0.996	0.990‐1.002
PCT	.241	1.420	0.790‐2.553
ESR	.051	1.041	1.001‐1.083
EOS	.561	0.305	0.006‐16.812
uRBC	.272	1.041	0.969‐1.118
BUN	.050	0.573	0.329‐1.000
Scr	.891	1.006	0.921‐1.100
ALT	.678	0.992	0.956‐1.029
AST	.219	1.062	0.965‐1.169
LDH	.066	0.992	0.984‐0.999
CK	.496	1.003	0.994‐1.012
CKMB	.839	1.006	0.952‐1.063

## 4 DISCUSSION

Asthma‐like symptoms is a common symptom among LRTIs cases. Frequent ALS onset may result in the airway injury, even the occurrence of asthma.^14^ Our study showed that lower age, male, inflammation, and allergic state were risk factors for ALS onset in LRTIs. These clinical and laboratory parameters were not independently associated with ALS risk, which suggested ALS was a multi‐factors disorder, comprehensive monitoring and assessment of these potential factors are needed in the prevention and therapy for ALS. Establishment of a preliminary risk prediction system for ALS onset is of great implications.

Lower age was noted to be associated with a higher risk of ALS among LRTIs, which may be attributed to that younger children were prone to present with respiratory tract infections due the lower immunity compared with older cases.^15^ On the other hand, age affected the association between obesity and asthma.^16^ Increased age resulted in the reduced influence of obesity on the asthma phenotype.^17^ Allergic reactions are likely to occur in the younger children due to the immaturity. Atopy reduced with the aging.^18^ These evidence may explain the higher incidence of ALS in younger children. We also noted that ALS cases had a higher boys/girls ratio, which may be due to the following facts: boys are likely to have a larger activity area, leading to an increased possibility of respiratory tract infections. Second, the androgen may limit the immune response, leading to the immunological disorders.^19^ Previous study also showed that boys had a stronger negative association between lung function and asthma.^20^

We observed that inflammatory markers, including WBC and PLT, were of predictive value for the risk of ALS onset among LRTIs. Chronic airway inflammation were involved in the development of asthma, inhibition of airway inflammation can lower the incidence of asthma and improve the clinical outcome.^21^ Neutrophils played an important role in the pathogenesis of allergic inflammation.^22^ On the other hand, a crosstalk existed between inflammation and oxidative stress, which was involved in the development of asthma.^23^ Notably, mycoplasma pneumoniae, respiratory virus infection and severe LRTIs did not affect the ALS incidence among LRTIs, which may be due to that ALS was a multiple‐factors disorder, single factor cannot influence the ALS risk independently.

Wheezing history and EOS were found to be associated with ALS risk among LRTIs. Allergic state was an important contributor to the asthma onset. For example, increased tree pollen counts in the spring were closely associated with asthma‐related emergency department visits.^24^ A close relationship existed between allergic rhinitis and asthma.^25^ These evidence supported the idea that allergic state was a risk factor for the risk of ALS onset among LRTIs. Interestingly, we found that the cardiac, liver, and kidney injury markers were not associated with the risk of ALS onset among LRTIs, which may be attributable to the facts that early stage of airway injury was mild, which did not affect the other organs. On the other hand, multivariate logistic regression analysis showed that the potential risk factors were not independently associated with the risk of ALS onset among LRTIs, which supported the idea that ALS was a multi‐factors disorder, multiple factors may interact to affect the development of ALS.^26^ Nevertheless, our findings had important clinical implications that more attention should be paid to the lower age, male, inflammation, and allergic state in LRTIs children.

Several limitations merit attention in our study. First, a cross‐sectional study design affected the long‐term observation of the association between the potential risk factors and ALS. On the other hand, we should also pay attention to the influence of ALS onset on the long‐term prognosis of LRTIs. Previous study also showed that ALS may be a presentation of antiphospholipid syndrome.^27^ Second, the interaction between the potential risk factors may affect the risk of ALS, although we performed a multivariate logistic analysis to identify whether the potential risk factors were independently associated with ALS risk. Gene‐environmental interactions were proved to be associated with asthma risk. For example, specific genotype combined with individual's proximity to roadways may influence the likelihood of asthma diagnosis and exacerbations.^28^ Further in‐depth analysis should focus on the interaction between these factors. Finally, due to lack of the environmental data, we did not investigate the influence of the environmental factors on the ALS risk. People from roadside colonies were likely to suffer from bronchial asthma.^29^ Further studies should be performed to analyze this issue deeply.

In conclusion, our investigation indicated that lower age, male, inflammation, and allergic state were risk factors for ALS onset in LRTIs. Comprehensive monitoring and evaluation of these factors may helpful for the ALS prevention.
